# Pigeons home faster through polluted air

**DOI:** 10.1038/srep18989

**Published:** 2016-01-05

**Authors:** Zhongqiu Li, Franck Courchamp, Daniel T. Blumstein

**Affiliations:** 1 School of Life Sciences, Nanjing University, Nanjing, 210023, China; 2 Department of Ecology and Evolutionary Biology, University of California, 621 Young Drive South, Los Angeles, 90095-1606, USA; 3 Center for Tropical Research, Institute of the Environment and Sustainability, La Kretz Hall, University of California Los Angeles, CA 90095, USA; 4 Ecologie, Systématique & Evolution, UMR CNRS 8079, Univ. Paris-Sud, Orsay Cedex, 91405, France

## Abstract

Air pollution, especially haze pollution, is creating health issues for both humans and other animals. However, remarkably little is known about how animals behaviourally respond to air pollution. We used multiple linear regression to analyse 415 pigeon races in the North China Plain, an area with considerable air pollution, and found that while the proportion of pigeons successfully homed was not influenced by air pollution, pigeons homed faster when the air was especially polluted. Our results may be explained by an enhanced homing motivation and possibly an enriched olfactory environment that facilitates homing. Our study provides a unique example of animals’ response to haze pollution; future studies are needed to identify proposed mechanisms underlying this effect.

Air pollution, especially haze pollution, has become a global environmental problem and the situation is much more serious in rapidly developing countries, such as China^1^,^2^, India^3^, Mexico^4^, etc. A large literature has documented deleterious effects of pollution on humans, such as increased incidences of heart disease, lung cancer, and high blood pressure^5^,^6^. Based on these risks, public health officials have suggested that people reduce or avoid outdoor activities to protect their health when the air is heavily polluted. However, much less is known about how non-human animals react to air pollution.

Due to their higher metabolic rate and special respiratory system, birds should be particularly sensitive to air pollution^7^,^8^ and, like in humans, air pollution has been documented to cause serious health problems for birds^7^,^9^. For instance, the accumulation of heavy metals and fine particles results in liver and lung damage^9^,^10^,^11^ and may affect flying ability. Moreover, birds’ navigation, which relies in part on visual or olfactory cues^12^,^13^, might also be affected by air pollution, because of the reduced visibility or chemical interference under conditions of heavy haze pollution^14^,^15^.

Homing pigeons are an ideal model system to study the effects of air pollution on bird behaviour. Pigeon navigation mechanisms are well-studied: pigeons use solar and geomagnetic cues as a compass to determine the direction to home, and they use olfactory and visual cues as a map to determine their position in space^16^,^17^. Thus, we might expect that pollution would negatively interfere with pigeon navigation and pigeons would both fly more slowly and be less successful at returning to their home roosts when flying through more polluted air. To test this hypothesis, we used a large data set generated by 415 pigeon races on the North China Plain, an area with China’s worst air pollution, to evaluate the effects of air pollution on pigeons’ homing performance.

## Results

**Figure 1:**
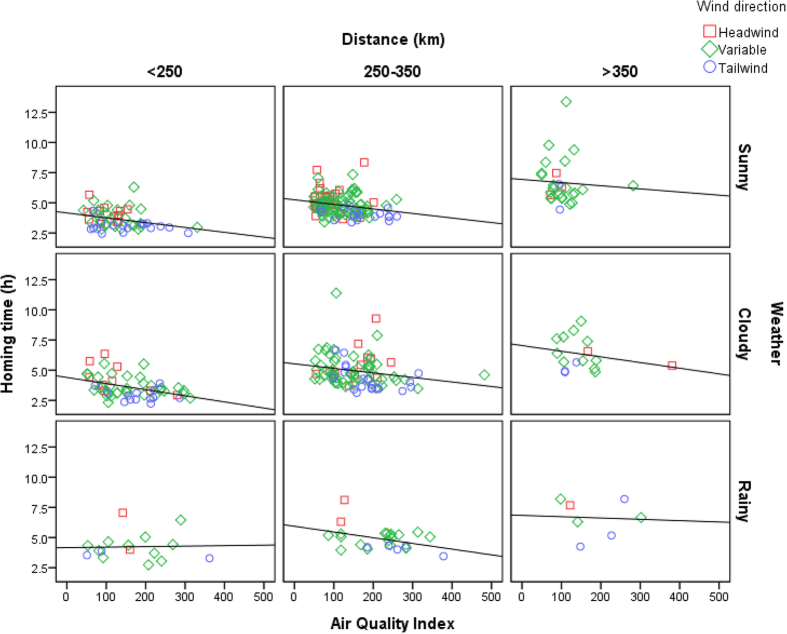
The relationship between AQI and observed average homing time (h) controlling for distance, weather and wind.

**Figure 2:**
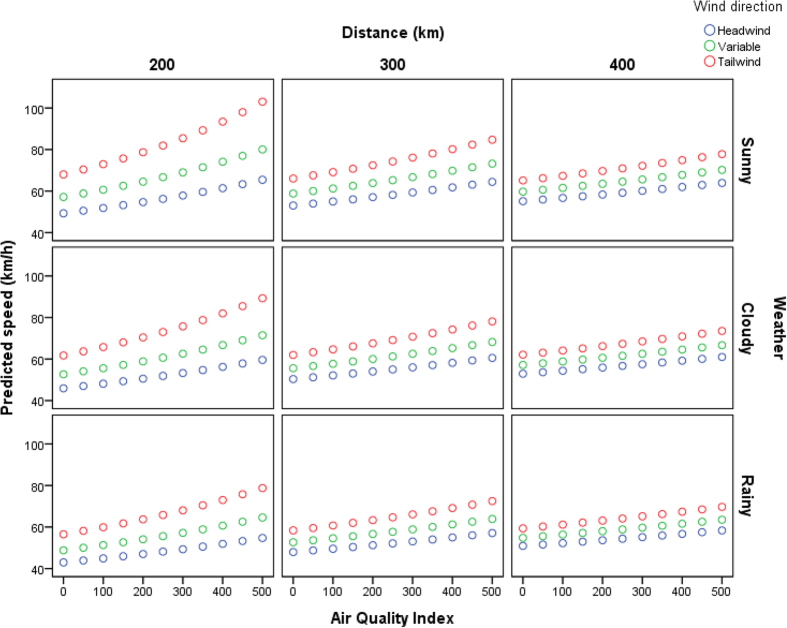
Predicted pigeon homing speed (km/h) with increasing AQI after controlling for distance, weather and wind.

Based on the availability of both environmental and racing data, we used each race as a unit of analysis and created a data set of 415 pigeon races on the North China Plain (Table 1). Four variables significantly affected pigeon homing time: average beeline distance (β ± SE = 0.016 ± 0.001, t = 34.104, P < 0.01), wind direction (β ± SE = −0.558 ± 0.081, t = −6.876, P < 0.01), weather conditions (β ± SE = 0.299 ± 0.070, t = 4.251, P < 0.01) and Air Quality Index (AQI) (β ± SE = −0.002 ± 0.001, t = −3.262, P < 0.01). Temperature (β ± SE = −0.003 ± 0.008, t = −0.394, P = 0.69) had no significant effects on homing time (Fig. 1). The model including all the above factors explained 96.4% of the total variance in pigeon homing time. Using our model’s parameter estimates to estimate homing speed, pigeons are predicted to increase their homing speed from 55.6 km/h when AQI = 0, to 68.2 km/h when AQI = 500; an increase of 22.7% when flying the median distance (300 km), under variable wind and through cloudy weather (Fig. 2).

The linear regression model of homing rate revealed significant effects of the intercept (β ± SE = 54.967 ± 6.366, t = 8.635, P < 0.01) and wind direction (β ± SE = 5.734 ± 1.506, t = 3.809, P < 0.01). Temperature (β ± SE = 0.096 ± 0.188, t = 0.511, P = 0.61), AQI (β ± SE = −0.018 ± 0.013, t = −1.363, P = 0.17), distance (β ± SE = −0.022 ± 0.014, t = −1.561, P = 0.12), and weather (β ± SE = 0.005 ± 1.351, t = −0.004, P = 0.99) had no effects on homing rate. This model explained only 4.4% of the total variance.

## Discussion

A number of studies have identified negative effects of anthropogenic features on the speed and success with which birds return home^18^,^19^. Indeed, we initially expected that pigeons would home more slowly due to the low visibility and potential negative health effects associated with air pollution. Contrary to our expectations, pigeons homed significantly faster when flying through more polluted conditions. We suggest two possible mechanisms to account for these unexpected findings: navigation ability and motivation^13^,^20^. It is generally accepted that pigeons use a two-step process to navigate, they use the sun and the geomagnetic field as a compass and they use visual and olfactory cues to create a map^13^,^16^. Could air pollution enhance pigeon visual and/or olfactory abilities, and by doing so explain the reduced homing time? Air pollution is usually associated with low visibility, particularly in North China where particulate matters are the main pollutants^15^,^21^. Decreased homing time under increased air pollution would suggest that the use of landmarks and visual cues for navigation might be important but not fundamental. This finding is consistent with previous studies that have shown that pigeons are able to home perfectly well from unknown sites where landmarks are unfamiliar^13^, even when flying with frosted lenses that impede vision^22^.

Olfactory cues have been shown to play an important role in avian navigation, and in pigeons it is probably a fundamental homing mechanism^13^,^16^. While air pollution cannot enhance vision, it might enhance olfactory navigation efficiency by providing supplemental olfactory cues to home. In Beijing, haze pollution is produced from several sources including coal burning, biomass burning, etc.^2^. As suggested by Wallraff and Andreae^23^, the majority of volatiles present in the air are of anthropogenic origin, and these organic and inorganic compounds could be potentially used for odour-based navigation. Further support for improved olfactory navigation under pollution requires identifying the precise chemical cues that pigeons use for navigation, and then demonstrating that these are associated with haze pollution. With respect to weather and wind direction, we found that pigeons homed faster on sunny days and when flying with a tailwind. This is likely due to both the availability of the sun compass and a boost in flight speed from a tailwind^24^,^25^. Wind direction also affected homing rate. Pigeons were more successful in returning to their home lofts when flying with a tailwind, likely because a tailwind provides mechanical support, thereby increasing homing speed and homing rate^24^,^25^.

Alternatively, decreased homing time under air pollution could be explained by an enhanced motivation to home; a possibility proposed several years ago that remains untested^13^. Prolonged exposure to polluted air could be detrimental to an individual’s health. For example, particulate matters^2^, the main pollutants in North China, have been shown to impede pigeon pulmonary function^11^. Thus, air pollution might be an indication of poor environmental quality, which might trigger rapid escape^26^,^27^. Motivation to home could also be enhanced if the reduced visibility under haze pollution^15^ increases predation risk because it interferes with the ability of pigeons to detect predators from afar^28^,^29^. Thus, by homing faster when flying through haze pollution, pigeons reduce the relative amount of time they are exposed to harmful or dangerous situations while away from the safety of their home roosts.

In conclusion, our results suggest that pigeons homed faster when flying through highly polluted air. We explained this finding by suggesting that pollution may enhance pigeons’ motivation to reduce exposure to health or predation risks associated with polluted air or the accompanying reduced visibility. An alternative hypothesis is that pollution enhanced olfactory navigation abilities, which provides more concentrated chemical volatiles that can be used by pigeons to build up an effective olfactory map. To discriminate between these alternative hypotheses, future studies should determine whether the reduced homing times result from increased flight speeds or from straighter flights. This could then help determine whether pollution increases the motivation to home (if the flight speed is increased, or resting time is decreased), and/or navigation performance (if flights are straighter and less tortuous). In addition, the possibility that pigeons could perceive a health risk associated with air pollution and fly faster as a result is an intriguing, new idea for environmental and human health, and would benefit from further testing in other non-human species.

## Materials and Methods

### Racing data

We obtained racing data from the public website of the Chinese Racing Pigeon Association (CRPA, http://www.crpa.net.cn/). For each race, data include city of the home loft and the city of release site, release time, arrival time of each pigeon, average beeline distance from the release site to the home lofts, number of pigeons released, and number of pigeons successfully returned. The homeward direction from the release site to the centre of the home lofts was calculated and categorised as North, Northeast, East, Southeast, South, Southwest, West, and Northwest. The distance (in km) of each race was calculated as the average of all returned pigeons.

Data focused the North China Plain, an area with the worst air pollution and for which the new Air Quality Index (AQI)^1^ – which integrates the most important haze source – PM2.5 – has been available since 2013. We focused on racing data from the fall of 2013 and 2014 because this is the time of year with the worst air quality^30^, and over half of the racing events are held during the fall. More importantly, pigeons behave differently between seasons^31^, and thus to eliminate variation, we focused on fall racing events. Since racing pigeons fly at an average of 60 km/h, and they are released mostly in the early morning, we eliminated races over 470 km to ensure that most pigeons potentially could return to their home lofts in the same day. The shortest race was 160 km long. Distances between different lofts were thus usually small compared to the race length, *i.e.*, within 30 km. With these criteria, we created a data set of 415 races (Supplemental file 1).

### Air Quality Index

We obtained Air Quality Index (AQI) data from the Data Centre of the Ministry of Environmental Protection of the People’s Republic of China (MEP, http://datacenter.mep.gov.cn/), or from related provincial or city meteorological departments. Based on established criteria (GB3095-2012), AQI is calculated for six major air pollutants separately: particle matter <10 microns in diameter (PM10), particle matter <2.5 microns in diameter (PM2.5), ground-level ozone level (O_3_), carbon monoxide (CO) level, sulphur dioxide level (SO_2_), and nitrogen dioxide level (NO_2_). An individual score is assigned to the level of each pollutant and the final AQI is the highest of those 6 scores. AQI values range from 0 to 500, and can be classified into six categories (Good: 0–50, Moderate: 51–100, Unhealthy for Sensitive Groups: 101–150, Unhealthy: 151–200, Very Unhealthy: 200–300, Hazardous: 301–500). In China, particulate pollution poses the greatest threat to human health in China, and AQI is well predicted by the concentrations of PM10 (r = 0.988, P < 0.01) and PM2.5 (r = 0.983, P < 0.01, Supplemental file 2).

Since all races were held in North China Plain, which is a broad plain without any geological obstructions, and the air quality is similar in adjacent cities^32^, we recorded the AQI at both the sites of release and the home lofts (if there were no AQI reports at either the release site or home lofts, we used AQI of the closest city; a distance <50 km). AQI levels at the release site and home lofts were positively correlated (r = 0.424, P < 0.01), so we used the average AQI to represent the pigeon’s air environment during a race.

### Meteorological variables

We obtained meteorological data from a public weather website (http://www.tianqihoubao.com/). We collated weather conditions, wind direction and ground air temperature (°C) at both the release and home lofts. Based on these data, we defined the weather conditions at the time of each race as: sunny, if both sites were sunny; cloudy, if either site was cloudy; and overcast or rainy (hereafter “rainy”), if either site was overcast or rainy. Precise information on wind speed was unavailable, so we focused on wind direction, which was classified into three categories: tailwind, when wind direction was the same as the direction of the birds’ flight at both release and home sites; headwind, a wind direction opposite to the birds’ flight directions at both sites; and variable, which included all other possible combinations of directions. We assumed that temperature increased smoothly from the lowest at sunrise (06:00 in September; 06:30 in October; 07:00 in November) to the highest at 14:00 and then decreased similarly. Then we calculated the average temperature of each race using the corresponding average homing times.

### Data analysis

We tested two hypotheses: under conditions of low visibility and olfactory interference associated with air pollution, pigeons would 1) increase their homing time and 2) decrease their homing rate (the percentage of pigeons successfully homed). To avoid a ratio-correlation problem that inevitably occurs when searching for relationships between speed (distance/time) and distance where distance appears on both sides of the equation^33^, we fitted a linear model using average homing time of each race as the dependent variable. Average beeline distance, weather, wind, AQI, temperature were defined as independent variables, and the intercept was set at 0. In the second linear model, we used the homing rate (percentage) as the dependent variable, and distance, weather, wind, AQI and temperature as independent factors. For categorical variables, we defined weather as sunny = 1, cloudy = 2, and rainy = 3, which indicated an increase of clouds cover, and wind as tailwind = 1, variable = 0, and headwind = −1, which indicated an effect of wind direction on flight difficulty. We did not include home city, homing direction and year in the final regression model, because we found no effects of city (F_6,397_ = 1.692, P = 0.12) or homing direction (F_3,397_ = 1.302, P = 0.27) on homing time in a preliminary analysis. Year (2013, 2014) explained significant variation in homing time (F_1,397_ = 9.885, P < 0.01), but since it was not the aim of our study to predict homing time in specific years, we excluded it from the final model. Individual pigeons vary in homing experience, and some pigeons are probably trained for uni-direction, which might bias their directional decision. Since we knew nothing about prior homing or training experience, we focused on homing time and homing rate, which are characteristics of a race, not an individual. Finally, we plotted the relationship between average actual homing speed and AQI, and we estimated the homing speed (beeline distance/homing time) using our regression model for three distances (200 km, 300 km, 400 km), under three weather conditions (sunny, cloudy, rainy), and three wind conditions (tailwind, headwind, variable). We reported coefficient values ± standard error. All analyses were conducted with SPSS 18.0.

### Ethics statement

Data on homing pigeon races were collected from public sources; no ethics approval was required for this study.

### Data availability

Data used for all analyses are available as electronic supplementary material.

## Additional Information

**How to cite this article**: Li, Z. *et al.* Pigeons home faster through polluted air. *Sci. Rep.* **6**, 18989; doi: 10.1038/srep18989 (2016).

## Figures and Tables

**Table 1:** Descriptive statistics of independent and dependent variables entered into the linear regression models of homing time and homing rate in racing pigeons.

Variables	Mean	SE	Minimum	Maximum	N
Weather					415
Sunny					205
Cloudy					162
Overcast or rainy					48
Wind					415
Tailwind					84
Variable					256
Headwind					75
AQI	143.86	3.48	42	482	415
Released pigeons	1591	54	74	7230	415
Returned pigeons	715	26	16	3358	415
Average beeline distance (km)	283.27	3.01	168.00	466.50	415
Homing time (h)	4.71	0.07	2.23	13.38	415
Temperature (°C)	15.46	0.24	1.00	26.82	415
Average home speed (km/h)	62.31	0.56	28.72	93.65	415
Homing rate	0.48	0.01	0.09	0.93	415
